# Efficient Color Conversion in Metal–Organic Frameworks Boosts Optical Wireless Communications beyond 1 GB/s Data Rate

**DOI:** 10.1021/jacs.4c16906

**Published:** 2025-02-11

**Authors:** Xin Zhu, Yue Wang, Tengjiao He, Simil Thomas, Hao Jiang, Osama Shekhah, Jian-Xin Wang, Tien Khee Ng, Husam N. Alshareef, Osman M. Bakr, Boon S. Ooi, Mohamed Eddaoudi, Omar F. Mohammed

**Affiliations:** † Center of Excellence for Renewable Energy and Storage Technologies, Division of Physical Science and Engineering, King Abdullah University of Science and Technology (KAUST), Thuwal 23955-6900, Kingdom of Saudi Arabia; ‡ Photonics Laboratory, Division of Computer, Electrical, and Mathematical Sciences and Engineering, King Abdullah University of Science and Technology, Thuwal 23955-6900, Kingdom of Saudi Arabia; § Functional Materials Design, Discovery, and Development Research Group (FMD3), Physical Science and Engineering Division, King Abdullah University of Science and Technology (KAUST), Thuwal 23955-6900, Kingdom of Saudi Arabia

## Abstract

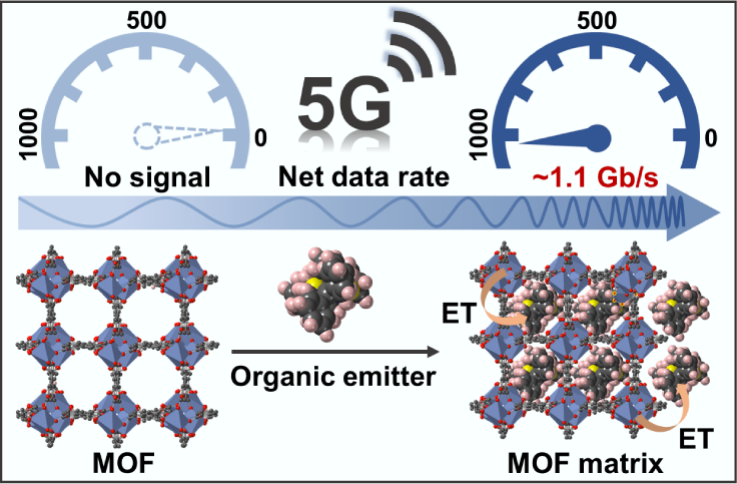

Efficient color converters are essential for achieving high −3-dB bandwidths and net data rates in optical wireless communications (OWCs). Here, we emphasize the significance of lanthanide-based metal–organic frameworks (MOFs) combined with an effective energy transfer strategy for developing high-performance color converters in OWC systems. In this approach, we successfully reduced the photoluminescence (PL) lifetime from 1.3 ms of the MOF to 4.6 ns of the MOF–chromophore composite, achieved through an efficient energy transfer process in the cavity and surface of the MOFs. This significant reduction in PL lifetime led to a dramatic increase in the −3-dB bandwidth, rising from less than 0.1 to 65.7 MHz. Most importantly, a net data rate of 1.076 GB/s was achieved, marking the first successful demonstration of lanthanide-based MOFs as color converters that facilitate data transmission rates exceeding 1 GB/s. Notably, both the −3-dB bandwidth and net data rate surpass those of most reported organic and inorganic materials, underscoring the exceptional potential of lanthanide-based MOFs when combined with an efficient energy transfer strategy. We believe this combination paves the way for further innovations in high-speed OWC technologies.

## Introduction

As the number of wireless communication devices grows exponentially, traditional radio frequency (RF)-based wireless data transmission networks face significant challenges, including a scarcity of spectrum resources and increasing network congestion.^1,2^ In this context, optical wireless communications (OWCs) present a promising alternative offering the key advantages of unlicensed and secure full-spectrum bandwidths from ultraviolet (UV) to infrared. This enables high-speed communication with enhanced security and confidentiality while avoiding electromagnetic pollution, RF radiation, and interference.^3−5^ In OWC systems, color-converting materials play a crucial role in transforming source signals from laser diodes or light-emitting diodes to achieve a high-color-rendering-index light emission, enabling high-performance large-area wide-field-of-view photodetection in underwater communication, vehicle-to-vehicle networks, and satellite links.^6−9^ Additionally, these materials determine the system’s bandwidth and data rates—key objectives in the field—spurring innovations aimed at enhancing communication efficiency and overall performance.^10,11^ However, traditional color-converting materials used in high-speed OWCs are, typically ceramic or perovskite-based, often containing environmentally harmful elements and requiring harsh synthesis conditions.^12−16^ These limitations have significantly hindered their potential for commercial applications. Therefore, identifying efficient material systems for OWCs that combine ease of synthesis, high stability, ecofriendliness, and capabilities for high bandwidth and data transmission speeds is both essential and highly challenging.

Lanthanide-based metal–organic frameworks (MOFs) represent an important class of luminescent materials, widely utilized in sensing, optoelectronic devices, and X-ray imaging scintillators, due to their tunable structures and unique optical properties.^17−21^ However, the long photoluminescence (PL) lifetimes of lanthanide-based MOFs limit their applications in OWC,^22,23^ where ultrashort PL lifetimes are essential to achieve high −3-dB bandwidth.^24,25^ Organic fluorescent chromophores, by contrast, typically exhibit very short PL lifetimes,^26^ making them ideal for OWC applications. However, their low PL efficiency in the deep red region significantly limits the data transmission rates. Thus, developing a strategy to combine the advantages of both lanthanide-based MOFs and organic chromophores could pave the way for a new generation of high-performance color converters for OWCs.^20,27^

Energy transfer is one of the most important processes in both natural and artificial systems, as it allows for the transformation of harvested energy from the donor to the acceptor while enhancing the reaction activity and PL efficiency of the energy acceptor.^28−32^ In this context, the intrinsic porosity of lanthanide-based MOFs promotes the integration of organic fluorescent emitters within the framework, thereby enhancing the donor–acceptor interactions. This strong donor–acceptor interaction could lead to high energy transfer efficiency, providing promising opportunities for designing high-performance color converters for OWC applications.^33−35^

**Figure 1:**
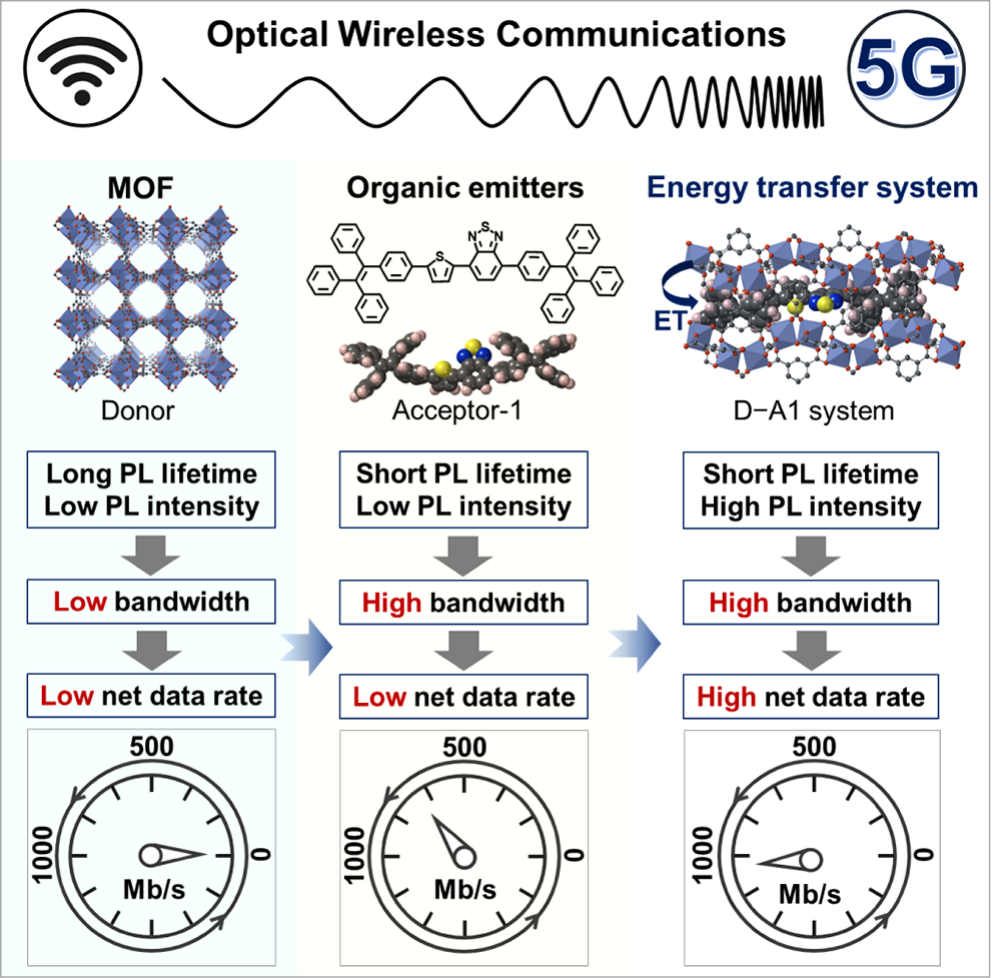
Illustration of the benefits of combining lanthanide-based MOFs with an efficient energy transfer strategy for the development of high-performance optical wireless communication applications.

Herein, we employed an efficient energy transfer strategy by utilizing a terbium-based MOF (namely, Tb-BTC MOF, BTC= benzene-1,3,5-tricarboxylic acid) as the energy donor and organic chromophores (A1 and A2) that emit in the deep red region as the energy acceptor. This approach facilitates the realization of high bandwidth and high-speed OWC applications. Specifically, the PL lifetime was effectively reduced from 1.3 ms for the MOF to 4.6 ns for the MOF–chromophore composite. This significant reduction resulted in an impressive enhancement of the −3-dB bandwidth, which increased from less than 0.1 to 65.7 MHz. Simultaneously, the net data rate increased dramatically from nearly 0 to 1.076 GB/s (Figure 1). Notably, both the −3-dB bandwidth and net data rate surpass those of most reported organic and inorganic materials, highlighting the significant potential of lanthanide-based MOFs combined with an efficient energy transfer strategy for developing high-performance color converters for the OWC applications.

## Results and Discussion

**Figure 2:**
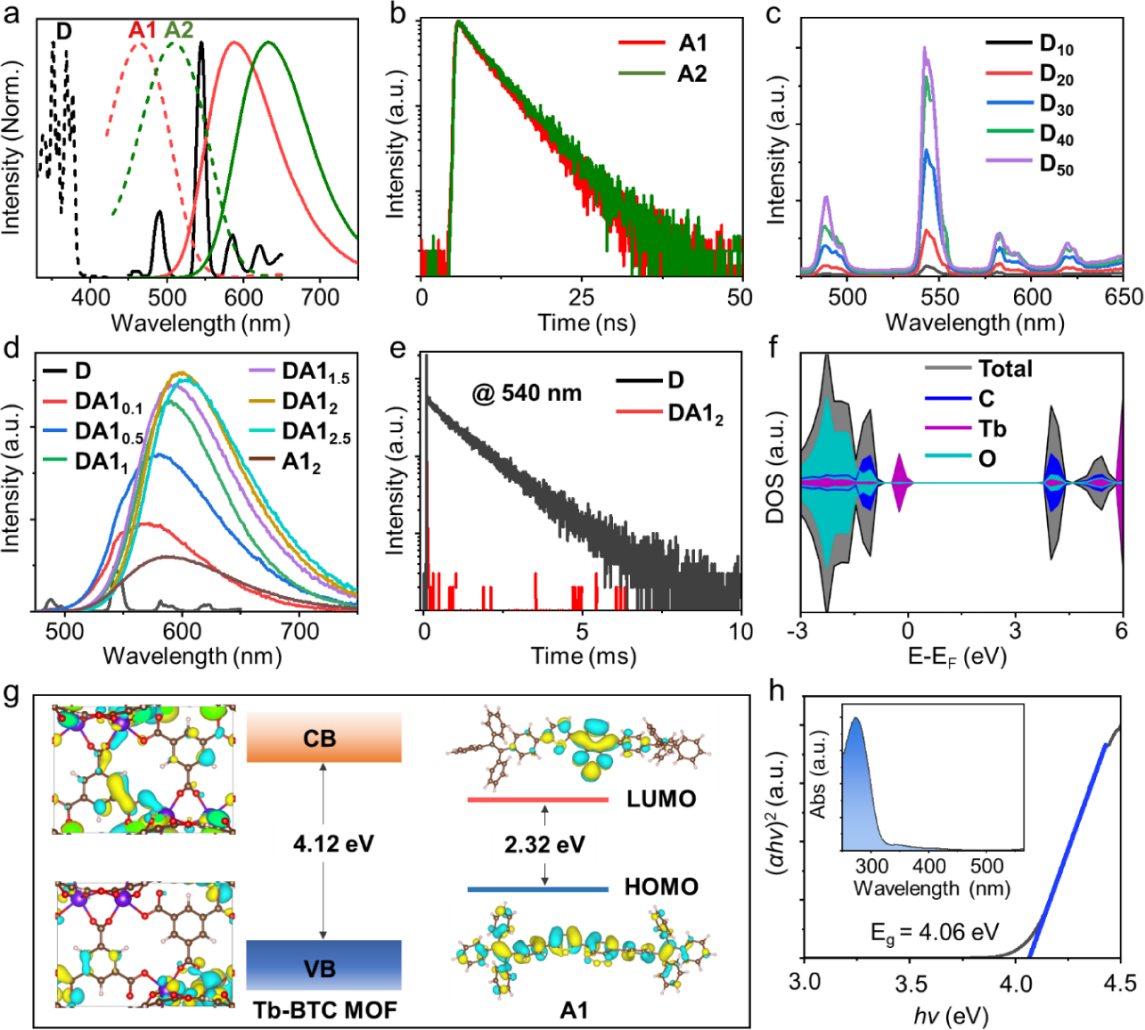
Photophysical properties and density functional theory (DFT) calculations. (a) Excitation (dashed line) and emission (solid line) spectra of Tb-BTC MOF (D) (black), along with the absorption (dashed line) and emission (solid line) spectra of A1 (red) and A2 (green). (b) The time-resolved emission decay profiles of A1 and A2 at 590 and 630 nm, respectively. (c) The emission spectra of the D_*n*_ doped in PMMA under 375 nm excitation, where *n* is the weight percentage of D. (d) The emission spectra of the DA1_*n*_ composite under 375 nm excitation, where *n* is the weight percentage of A1, with the concentration of D fixed at 50 wt %. (e) Time-resolved emission decay profiles of D and DA1_2_ at 540 nm. (f) Projected density of states (PDOS) of Tb-BTC MOF. (g) Electronic charge densities for the conduction band minimum (CBM) and the valence band maximum (VBM) of Tb-BTC MOF, and energy transfer diagram of the DA1 system. (h) The optical band gap of D (the inset shows the UV–vis absorption spectra).

The lanthanide-based MOF Tb-BTC was synthesized using benzene-1,3,5-tricarboxylic acid (H_3_BTC) as the organic linker. In this structure, terbium ions (Tb^3+^) function not only as the metal center but also as the emission source. The structure of the Tb-BTC MOF was characterized through powder X-ray diffraction (PXRD) patterns and Fourier transform infrared spectroscopy (FTIR) measurements (Figures S1 and S2). The PXRD results demonstrated excellent agreement with the simulated data,^36^ confirming the crystalline structure of the MOF. Similarly, the FTIR spectra showed strong alignment with previously reported data,^37^ further validating the successful coordination between the Tb^3+^ ions and the carboxylate groups. Tb-BTC MOF (D) displays a well-defined excitation spectrum between 330 and 430 nm and an emission band ranging from 450 to 650 nm, featuring an intense peak at 540 nm (Figure 2a). Meanwhile, the organic chromophores A1 and A2 were selected as energy acceptors due to their long wavelength emission and short PL lifetime properties. Both A1 and A2 have broad absorption spectra within the UV–visible range, with absorption maxima at 465 and 510 nm, respectively. Additionally, these chromophores display broad emission bands in the red region, with A1 emitting at 590 nm and A2 emitting at 630 nm (Figure 2a). A particularly advantageous feature of A1 and A2 is their short PL lifetimes of 4.6 and 4.7 ns, respectively (Figure 2b). These short lifetimes are critical for achieving the rapid response times needed in high-performance OWC color converters.

To fabricate the energy transfer system using Tb-BTC MOF as the energy donor and A1 or A2 as the energy acceptor for OWC applications, we first optimized the doping concentration of the Tb-BTC MOF in a poly (methyl methacrylate) (PMMA) matrix (Figure 2c). The concentration was varied from 10 to 50 wt %, during which we observed a gradual increase in PL intensity with higher MOF concentrations. The concentration was limited to 50 wt % to maintain the film’s quality, as higher concentrations risked compromising the mechanical strength of the film for optimal OWC performance. It is worth noting that incorporating the benzothiadiazole unit into A1 or A2 results in a redshift in the emission spectra, distinguishing them from the donor. Moreover, these two chromophores exhibit excellent energy alignment with the MOF, ensuring efficient energy transfer from the MOF to the acceptor. Additionally, the molecular sizes of A1 and A2 are well-matched to the MOF’s pore size, enhancing donor–acceptor interactions and improving energy transfer efficiency. As shown in Figures 2d and S3a, increasing the concentration of energy acceptors (DA1_*n*_ and DA2_*n*_, where *n* represents the weight percentage (wt %) of A1 or A2, with the concentration of D fixed at 50 wt %) leads to a progressive quenching of the PL spectra of D, which nearly disappears, while the PL intensity of the acceptors correspondingly increases. This trend is also reflected in a substantial decrease in the PL lifetime of D, as depicted in Figures 2e and S3b. Taking A1 as an example, when its concentration reaches 2 wt %, the D’s lifetime at 540 nm decreases from 1.3 ms to 9.6 μs (Figure 2e), resulting in an energy transfer efficiency (ε) of 99% (as shown in eqs 1 and 2).
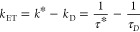
1
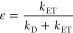
2where τ_D_ and τ* are the photoluminescence lifetimes of the donor in the absence and presence of the acceptor, respectively. It is worth pointing out that the stronger intermolecular π–π stacking at higher loading concentrations of A1 or A2 leads to the redshift in the emission spectra, in good agreement with findings reported previously.^38,39^ To validate this mechanism, we calculated the lowest unoccupied molecular orbital (LUMO)–highest occupied molecular orbital (HOMO) of A1 and A2 in their monomeric and dimeric forms (Figure S4). The results demonstrate that the dimer exhibits a slightly reduced LUMO–HOMO energy gap, confirming the aggregation-induced emission redshift.

To further elucidate the underlying energy transfer mechanism, density functional theory (DFT) calculations were conducted. The density of states (DOS) analysis for the Tb-BTC MOF, projected onto the atomic components, reveals that the valence band maximum (VBM) and the conduction band minimum (CBM) are localized on the Tb atoms (Figure 2f,g), confirming that the primary emission originates from the metal center. Additionally, calculations of the electronic band structure for the Tb-BTC MOF and A1 indicate a direct band gap of 4.12 and 2.32 eV, respectively (Figure 2g), which closely aligns with the experimentally measured value of 4.06 and 2.37 eV (Figures 2h and S5), validating the theoretical model. Moreover, the CBM of Tb-BTC MOF is higher than the LUMO levels of acceptors A1 and A2, while the VBM of Tb-BTC MOF is positioned lower than the HOMO levels of A1 and A2 (Figures 2g and S6). These specific energy level alignments suggest favorable conditions for efficient energy transfer from the donor to the acceptors.

**Figure 3:**
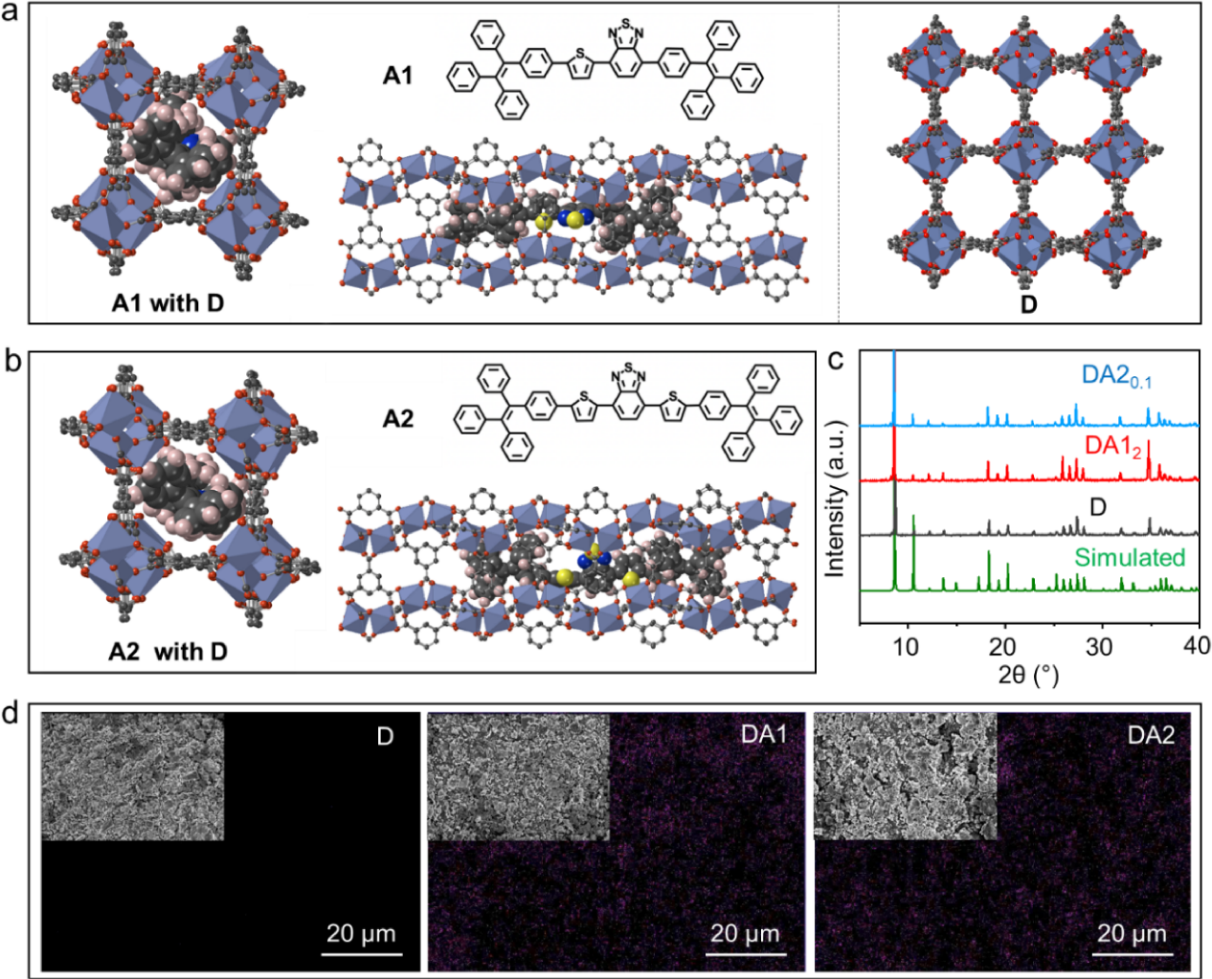
Structure characterization. Schematic showing the molecular structures and geometrical constraints of (a) DA1 and (b) DA2 with A inside D. Tb, O, C, N, and S atoms are shown in purple, red, gray, blue, and yellow, respectively. H atoms are omitted. (c) Comparison of powder X-ray diffraction (PXRD) patterns of D, DA1_2_, and DA2_0.1_ with the simulated patterns of D. (d) SEM images and EDS elemental mappings of S element for D, DA1, and DA2.

To explore how donor–acceptor interactions drive efficient energy transfer between the Tb-BTC MOF and the organic chromophores, structural simulations were performed (Figure 3a,b). The Tb-BTC MOF features a cavity size of approximately 11 Å, which could effectively encapsulate the acceptor molecules A1 and A2, enabling close contact and facilitating strong donor–acceptor interactions, ultimately resulting in efficient energy transfer. Furthermore, the acceptor molecules can also adsorb onto the surface of the Tb-BTC MOF, further enhancing the donor–acceptor interactions due to increased contact points (Figure S7). Moreover, the structural analysis confirmed that the incorporation of A1 or A2 into Tb-BTC MOF did not disrupt the MOF’s framework. This is supported by the PXRD patterns (Figures 3c and S8), where the diffraction peaks of the doped MOF align perfectly with the simulated patterns, indicating that the crystalline structure of the Tb-BTC MOF remains intact after doping. In addition, energy-dispersive X-ray spectroscopy (EDS) elemental mapping reveals a uniform distribution of sulfur (S)—a characteristic element of the acceptor molecules—throughout the energy transfer system, confirming the successful incorporation of A1 or A2 within the Tb-BTC MOF matrix (Figures 3d and S9). This uniform distribution highlights the effectiveness of the doping process and further supports the development of a robust energy transfer system.

**Figure 4:**
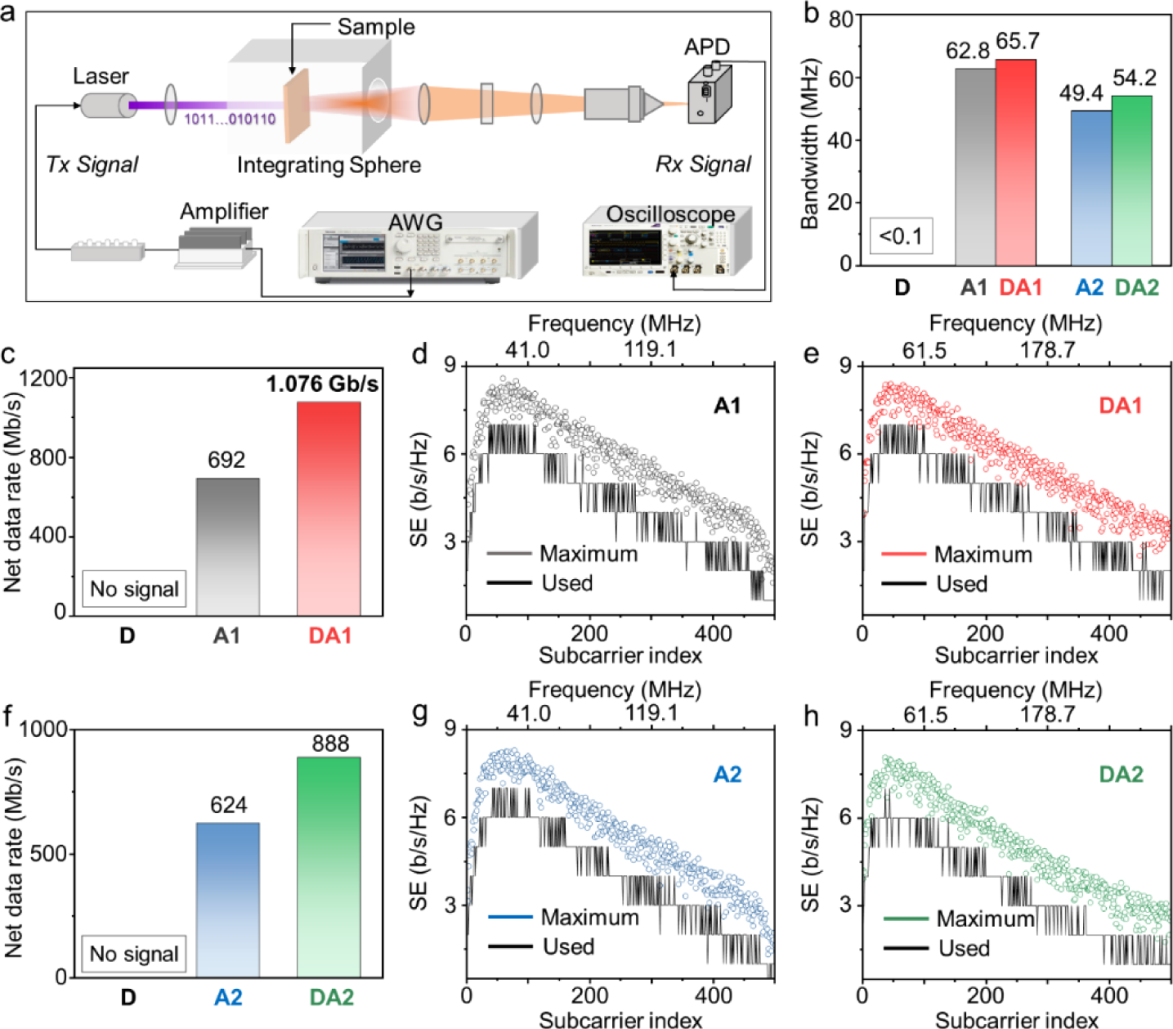
Optical modulation measurements utilizing DC-biased optical orthogonal frequency-division multiplexing (DCO-OFDM). (a) Schematic representation of the OWC channel. (b) The −3-dB bandwidth of A1_2_, DA1_2_, A2_0.1_, and DA2_0.1_ (the bandwidth of D exceeds the system’s frequency limit). (c) Net data rate comparison between D, A1_2_, and DA1_2_. The spectral efficiency (SE) of (d) A1_2_ and (e) DA1_2_ during DCO-OFDM implementation. (f) The net data rate comparison between D, A2_0.1_, and DA2_0.1_. The SE of (g) A2_0.1_ and (h) DA2_0.1_ during DCO-OFDM implementation (no signal bits of D could be transferred due to insufficient bandwidth and SNR).

Due to the strong encapsulation and adsorption properties of Tb-BTC MOF for organic chromophores A1 and A2, efficient energy transfer reduced the system’s PL lifetime from D’s 1.3 ms to the A’s 4.6 ns while significantly enhancing the acceptor’s PL intensity. This energy transfer mechanism thus fulfills the essential requirements for a high-performance OWC system: short PL lifetime and high PL intensity. To demonstrate the feasibility of the developed energy transfer strategy in OWC applications, the performances of the Tb-BTC MOF, A1_2_, A2_0.1_, DA1_2_, and DA2_0.1_ were evaluated in the OWC channel illustrated in Figure 4a. The samples were placed inside an integrating sphere and illuminated using a 375 nm laser diode. The emitted fluorescence was collected and converted into an electrical signal by an avalanche photodetector (APD). A vector network analyzer (VNA) was used to modulate the laser current, and the output signal from the APD was analyzed to obtain the frequency responses across various frequencies. By applying sinusoidal alternating current (AC) signals within the 300 kHz to 100 MHz range, the −3-dB modulation bandwidths for DA1 and DA2 were determined to be 65.7 and 54.2 MHz, respectively (Figures 4b and S10). The D–A system’s high modulation bandwidths slightly exceed those of pure acceptors (62.8 MHz for A1_2_ and 49.4 MHz for A2_0.1_) and are significantly greater than that of the pure donor, which has a bandwidth below 0.1 MHz of the system’s frequency limit (Figure 4b). Typically, the bandwidth of luminescent materials is influenced by their PL lifetime, with shorter PL lifetimes generally leading to wider bandwidths. However, this is not the only determining factor. Other parameters, including the material’s composition, emission mechanisms, and interactions with the surrounding environment, also play significant roles in shaping the bandwidth. In this context, the differing compositions of DA1 and DA2, along with variations in their energy transfer processes, may explain the slight differences in the bandwidth observed between pure A1 and A2.

Moreover, direct-current-biased optical orthogonal frequency-division multiplexing (DCO-OFDM) modulation was performed to further validate the proof of concept that energy-transfer-based MOF materials are viable candidates for color-conversion phosphors in OWC links. To evaluate the channel capacity, a uniform 4-quadrature amplitude modulation (4-QAM) OFDM test signal was transmitted through the 375 nm laser at an operating current of 100 mA, corresponding to 827.6 mW/cm^2^ irradiance on the sample (Figure 4a). The signal-to-noise ratio (SNR) was then calculated by using the error vector magnitude (EVM) in conjunction with the channel capacity formula log_2_(1+SNR). After adaptive bit allocation (Figure 4c - h) and power loading (Figures S11 and S12), the net data rates were achieved for DA1_2_ and DA2_0.1_ of 1.076 GB/s and 888.01 MB/s, respectively, which exhibit remarkable enhancement compared to the performance of Tb-BTC MOF alone (no signal could be transferred due to insufficient bandwidth and SNR), as summarized in Figure 4c and 4f. Note that the exceptional performance of loading A1 can be attributed to several key factors. First, A1 has a molecular size slightly smaller than that of A2, which promotes more favorable interactions with the MOF, leading to enhanced energy transfer efficiency. Second, A1 features a shorter PL lifetime, resulting in a broader emission bandwidth and, consequently, an improved net data rate. Finally, the photodetector utilized in the OWC applications exhibits greater sensitivity within the emission wavelength range of A1, further contributing to its superior performance. The bit error rate (BER) values for DA1 and DA2 are over 3.3 × 10^–3^ and 3.7 × 10^–3^, respectively, which are below the forward error correction (FEC) limit of 3.8 × 10^–3^. Figure S13 shows the constellation diagrams of all QAM orders used up to 128-QAM. These results significantly outperform those of most MOF materials (Table 1) and many commercially available ceramic, perovskite, and organic materials.^40−44^ Furthermore, MOFs are known for their robustness under environmental stresses including moisture, heat, and UV radiation. This makes them more reliable in outdoor and harsh environments when compared to metal-halide perovskites.^45,46^ These findings further validate the significant potential of the energy transfer strategy in the development of innovative, high-performance color converters specifically designed for the use in OWC applications.

**Table 1:** Performance Comparison of Color-Converting Materials Based on an MOF/COF for Optical Wireless Communications

Materials	Net data rate	–3-dB Bandwidth	Ref.
RhB@Al-DBA-MOF	3.6 MB/s	3.6 MHz	(42)
Zr-TCBPE-MOL	3.5 MB/s	1.7 MHz	(34)
PIM-1@NU-1000	215 MB/s	78.3 MHz	(33)
Hf-BT-fcu-MOF	362 MB/s	118.5 MHz	(35)
Hf-BI-fcu-MOF	363 MB/s	62.1 MHz	(35)
Zr-BT-fcu-MOF	357 MB/s	111.5 MHz	(35)
Zr-BI-fcu-MOF	303 MB/s	150.0 MHz	(35)
AIE-COF	825 MB/s	200 MHz	(9)
DA1	**1.076** GB/s	65.7 MHz	This work
DA2	**888** MB/s	54.2 MHz	This work

## Conclusion

In this study, we present a groundbreaking approach that integrates encapsulated organic emitters within the cavities of lanthanide-based MOFs, achieving remarkably efficient energy transfer and creating high-performance color converters for OWC applications. By using this unique method, we successfully reduced the PL lifetime from 1.3 ms of the Tb-BTC MOF to an impressive 4.6 ns of the MOF–chromophore composites, resulting in a dramatic increase in the −3-dB bandwidth from less than 0.1 to 65.7 MHz. Even more compelling, we achieved a net data rate of 1.076 GB/s, marking the first demonstration of lanthanide-based MOFs facilitating data transmission rates exceeding 1 GB/s. Notably, both the −3-dB bandwidth and net data rate surpass those of most existing organic and inorganic materials, highlighting the exceptional potential of lanthanide-based MOFs coupled with innovative energy transfer strategies. This synergy not only represents a major leap forward in high-speed OWC technology but also opens new avenues for future research and development in the fields of both MOF and OWC.

## Supporting Information Available

Details of MOF synthesis and film fabrication, computational methods, material characterizations, figures detailing SEM images, photophysical performance, and OWC performance (PDF)
