# Supplementary material for: Efficient Color Conversion in Metal–Organic Frameworks Boosts Optical Wireless Communications beyond 1 GB/s Data Rate
Source: J Am Chem Soc. 2025 Feb 11;147(8):6805–12. doi: 10.1021/jacs.4c16906 (PMC11869274; doi:10.1021/jacs.4c16906)
Supplement: Supplementary file 1 — ja4c16906_si_001.pdf [file ja4c16906_si_001.pdf]

Supporting Information

Efficient Color Conversion in Metal-Organic Frameworks Boosts Optical Wireless Communications Beyond 1 Gb/s Data Rate

Xin Zhu[†], Yue Wang[‡], Tengjiao He[§], Simil Thomas[†], Hao Jiang[§], Osama Shekhah[§], Jian-Xin Wang[†, §], Tien Khee Ng[‡], Husam N. Alshareef[†], Osman M. Bakr[†], Boon S. Ooi[‡, *], Mohamed Eddaoudi[§, *], Omar F. Mohammed[†, *]

[†]Center of Excellence for Renewable Energy and Storage Technologies, Division of Physical Science and Engineering, King Abdullah University of Science and Technology (KAUST), Thuwal 23955-6900, Kingdom of Saudi Arabia

[‡]Photonics Laboratory, Division of Computer, Electrical, and Mathematical Sciences and Engineering, King Abdullah University of Science and Technology, Thuwal 23955-6900, Kingdom of Saudi Arabia

[§]Functional Materials Design, Discovery, and Development Research Group (FMD3), Physical Science and Engineering Division, King Abdullah University of Science and Technology (KAUST), Thuwal 23955-6900, Kingdom of Saudi Arabia

*Corresponding Authors: Boon S. Ooi, Email: boon.ooi@kaust.edu.sa; Mohamed Eddaoudi, Email: mohamed.eddaoudi@kaust.edu.sa; Omar F. Mohammed, Email: omar.abdelsaboor@kaust.edu.sa

Materials and Methods

All chemicals were purchased from commercial suppliers (Sigma-Aldrich) and used without further purification. Chromophores A1 and A2 were purchased from Amadis Chemical, with their synthesis methods detailed in the reported literature.[1] UV-Vis absorption measurements were performed with PerkinElmer Lambda 950 UV/VIS Spectrometer. PerkinElmer LS45 Photoluminescence spectrometer having 450 W Xenon lamp was used for steady-state photoluminescence studies. Powder X-ray diffraction (PXRD) patterns were collected by Bruker D8 ADVANCE diffractometer for Cu Kα radiation (λ = 1.5406 Å). Scanning Electron Microscope (SEM) images were acquired using a Magellan 400 scanning electron microscope and FEI™, QUANTA 200 3D with tungsten cathode as an electron source.

Synthesis of Tb-BTC MOF. $Tb(NO_3)_3 \cdot xH_2O$ and H_3BTC, in a molar ratio of 3:1, were dissolved in 8 mL of DMF and 2 mL of H_2O using ultrasonication for 5 minutes at room temperature. The resulting mixture was then sealed and heated in a water bath at 70 °C for 12 hours. The as-synthesized Tb-BTC MOF microcrystals were subsequently rinsed several times with DMF and ethanol, and then dried in an oven at 70 °C for 24 hours. The simulated PXRD pattern of the Tb-BTC MOF was calculated from the reported CIF file.[2]

Preparation of the films. The preparation began by dispersing 50 mg of Tb-BTC MOF and the corresponding organic chromophores in 0.6 mL of chloroform. The mixture was sonicated for 5 minutes, followed by the addition of 50 mg of poly(methyl methacrylate) (PMMA). After an additional 5 minutes of sonication, the mixture was shaken on a shaker for 5 hours to ensure thorough mixing of all materials. The resulting viscous solution was then carefully coated onto quartz plates to create films for UV and visible light communication-related measurements. To ensure uniformity, the films were covered with a beaker during the solvent evaporation process.

Computational methods. The structure models of DA1 and DA2 were built with Materials Studio 2019. Geometry optimizations of A1, A2 molecules and Tb-BTC MOF were performed using the Vienna ab initio simulation package (VASP)[3] at the generalized gradient approximation level using Perdew–Burke–Ernzerhof functional. Van der Waals dispersion effects were considered at the DFT-D3 level. A plane-wave cutoff of 400 eV was applied, and a Γ-centered k-mesh with a k-spacing of 0.25 $Å^{-1}$ was used to sample the Brillouin zone for the self-consistent field (SCF) calculations. Structure relaxations were performed until the atomic forces were smaller than 0.02

eV/Å. Final single-point calculations at the HSE06 level are used to calculate the density of states and wavefunctions.

Small-signal frequency response. A 375-nm laser diode (LD) (Nichia, NDU4116) served as the transmitter. The diode was housed in a laser diode mount (Thorlabs, LDM56F/M), which includes an integrated thermo-electric cooler, temperature controller, and bias-tee. The operating temperature of the LD was maintained at 21 °C. The RF signal at different frequencies was generated by a vector network analyzer (VNA) (Agilent Technologies, E5061B) to modulate the driving current of LD. The modulated laser beam was collimated and guided into the integrating sphere to excite the samples. Then, the scattered output light was focused through a series of aspheric condensers and an objective lens. To prevent any unabsorbed photons from the excitation source from reaching the photodetector, a 400-nm long-pass (LP) filter (Thorlabs, FELH0400) was positioned between the two aspheric condensers. A silicon-based avalanche photodetector (Thorlabs, APD430A2/M) with an active diameter of 0.2 mm and an output –3-dB bandwidth of up to 400 MHz was mounted after the objective lens as a receiver. The electrical signal from the APD was analyzed by the VNA to obtain the frequency response information.

The key advantage of our OWC system lies in its ability to convert UV light into various visible wavelengths, enabling tunable data transmission properties through the use of different color converters. This work utilizes an energy transfer strategy to optimize the MOF's data transmission performance. Specifically, incorporating A1 or A2 into the MOF efficiently converts UV photons absorbed by the MOF into longer-wavelength visible light emitted by A1/A2, thereby enhancing data transmission efficiency. To ensure a meaningful comparison between pure A1/A2 and the DA composite, we used a 375 nm excitation source. Under these conditions, the addition of the MOF as an energy donor increased the overall data transmission performance of A1/A2.

DC-biased optical orthogonal frequency-division multiplexing (DCO-OFDM). On the transmitter side, 2^{16}-1 pseudorandom binary sequence (PRBS) was generated and converted into an array with the dimension defined by the number of used subcarriers (500) and the number of OFDM symbols (150). After 4-quadrature amplitude modulation (QAM), Hermitian symmetry was imposed to ensure that the output of the inverse fast Fourier transform (IFFT) is real-valued. Then, a cyclic prefix number of 10 was added to minimize inter-symbol interference (ISI) and approximate circular convolution with the impulse response of the channel, resulting in

multiplication in the frequency domain, which simplifies applying single-tap equalization based on channel estimation from the training symbols. The parallel sequence is then converted back into a serial sequence before transmission through the AWG. On the receiver side, after synchronization, the signal is converted to a parallel sequence, and the cyclic prefix is removed before performing an FFT. The symbols added for the Hermitian symmetry are then removed, and single-tap equalization is applied. The QAM symbols are demodulated, and the resulting data are converted to a serial sequence for comparison with the transmitted signal to determine the BER. The gross data rate was calculated, and the net data rate was obtained by removing the training symbols and 7% overhead needed for forward error correction (FEC) from the gross data rate to ensure that the BER is below the FEC BER limit of 3.8×10^{-3}.

After removing the thiophene rings, the chromophore's emission blue shifts to 540 nm, which is unsuitable for the energy transfer system with the MOF as they share the same emission wavelength. However, we measured the data rate for this chromophore alone, which is approximately 470 Mb/s, slightly lower than that of A1 and A2.

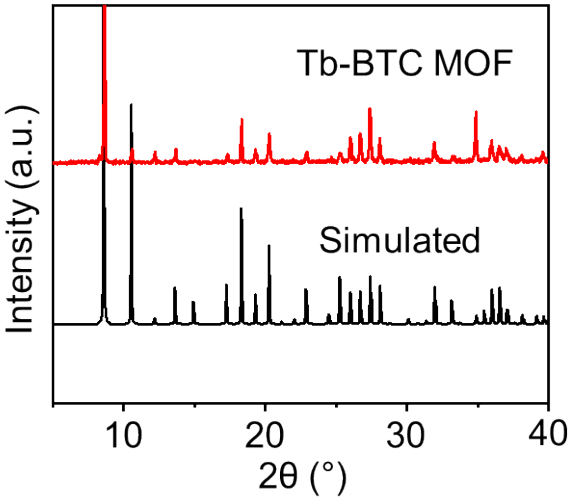

Figure S1. Powder X-ray diffraction (PXRD) pattern of the synthesized Tb-BTC MOF compared with the simulated pattern. The simulated PXRD pattern of the Tb-BTC MOF was calculated from the reported CIF file.[2]

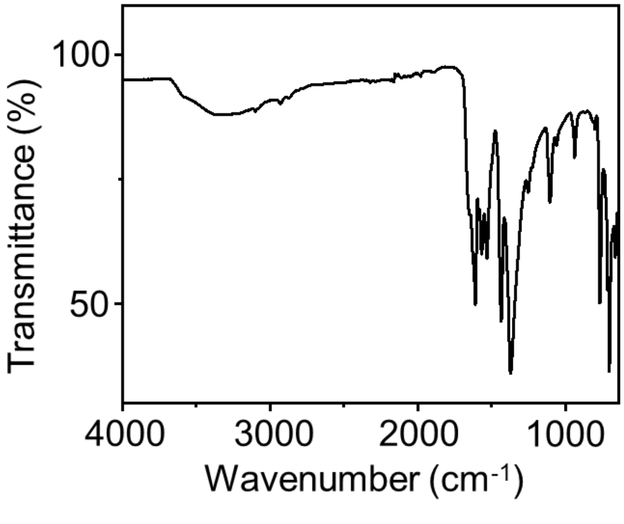

Figure S2. The FTIR spectrum of the synthesized Tb-BTC MOF.

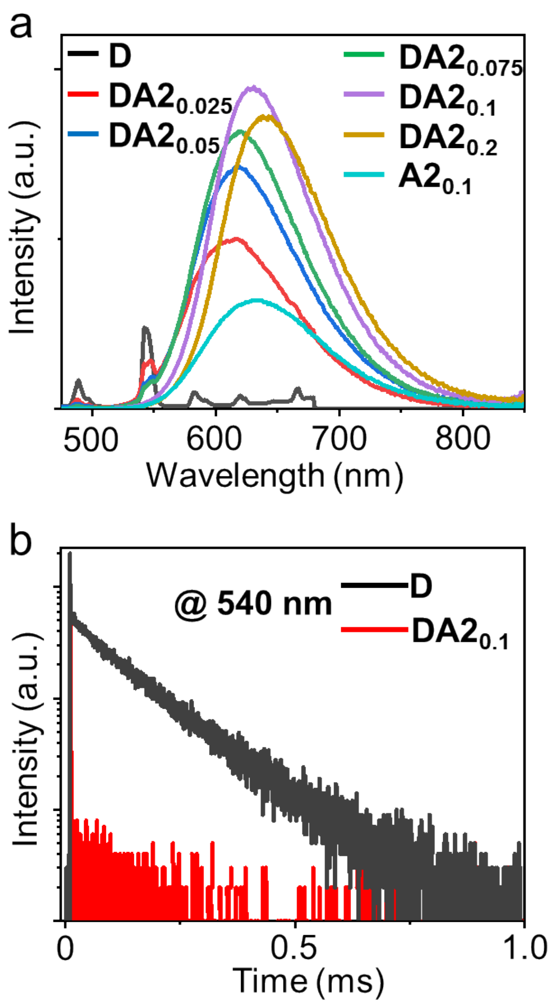

Figure S3. (a) The emission spectra of the DA2$_n$ composite in a film state under 375 nm excitation, where n is the weight percentage of A2. (b) The time-resolved emission decay profiles of D and DA2$_{0.1}$.

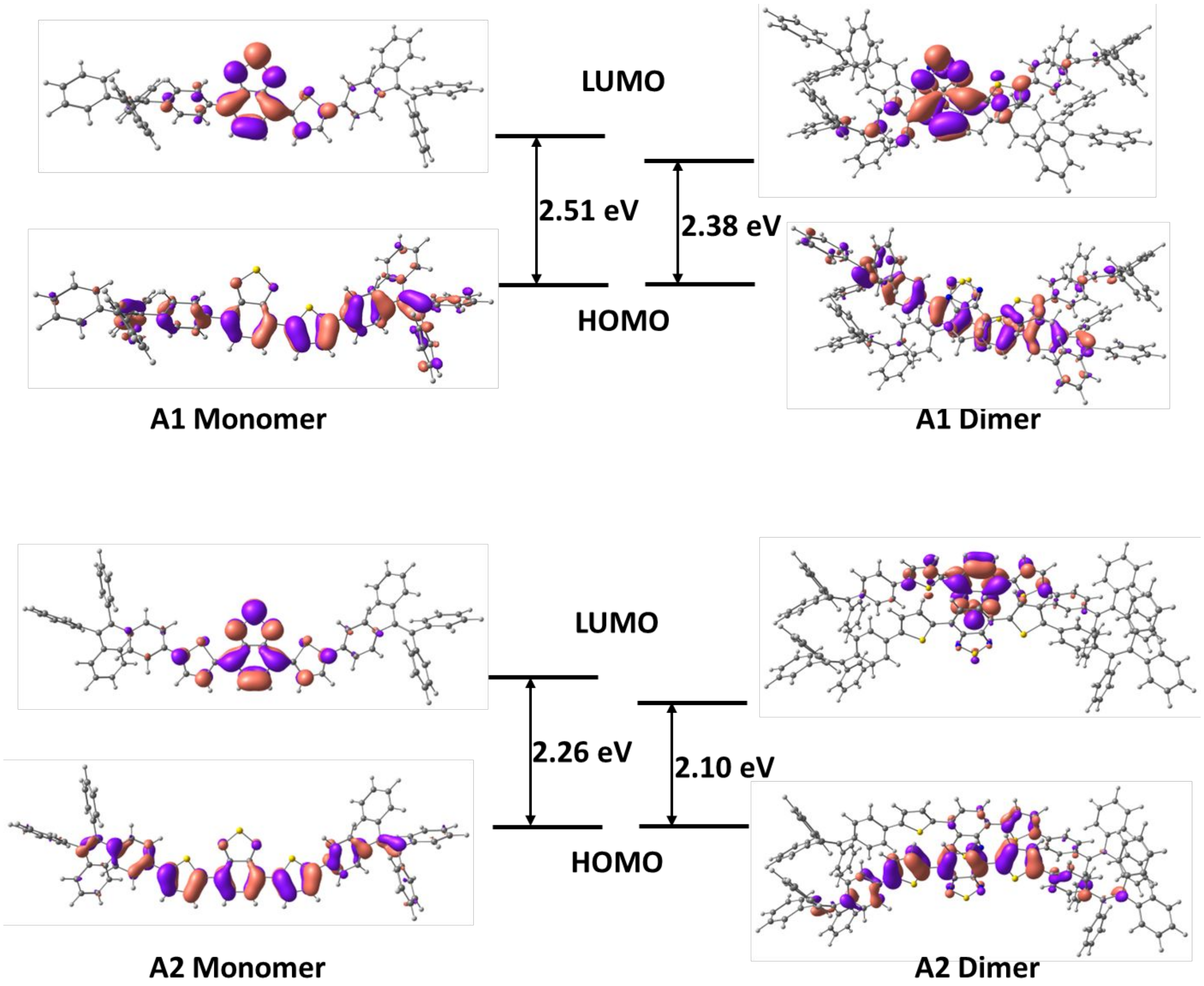

Figure S4. Calculated LUMO-HOMO gap of A1 and A2 in their monomeric and dimeric forms.

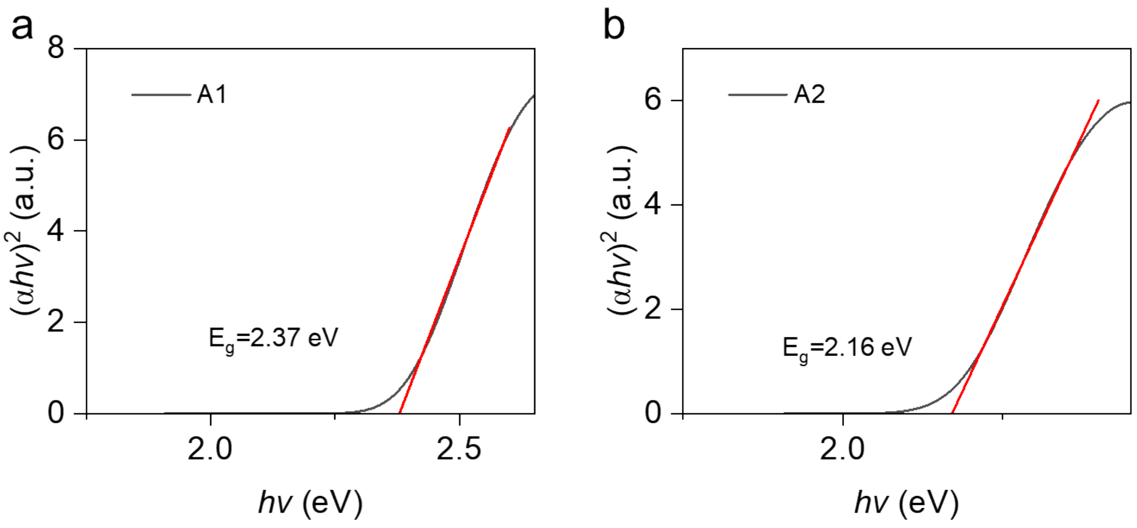

Figure S5. The optical band gap of (a) A1 and (b) A2.

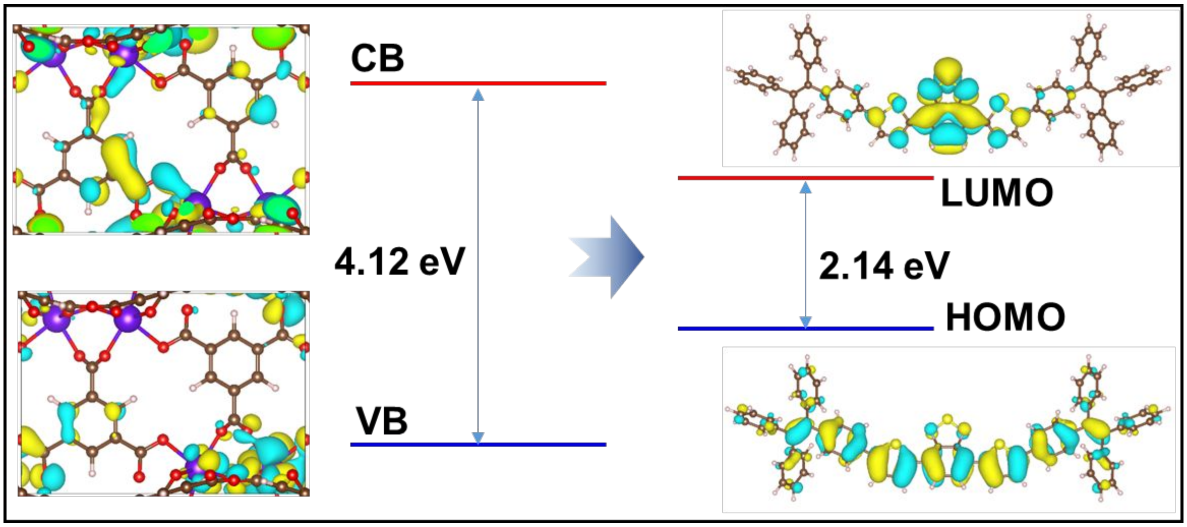

Figure S6. Energy transfer diagram within the DA2. The DFT calculations were performed at the HSE06 level of theory.

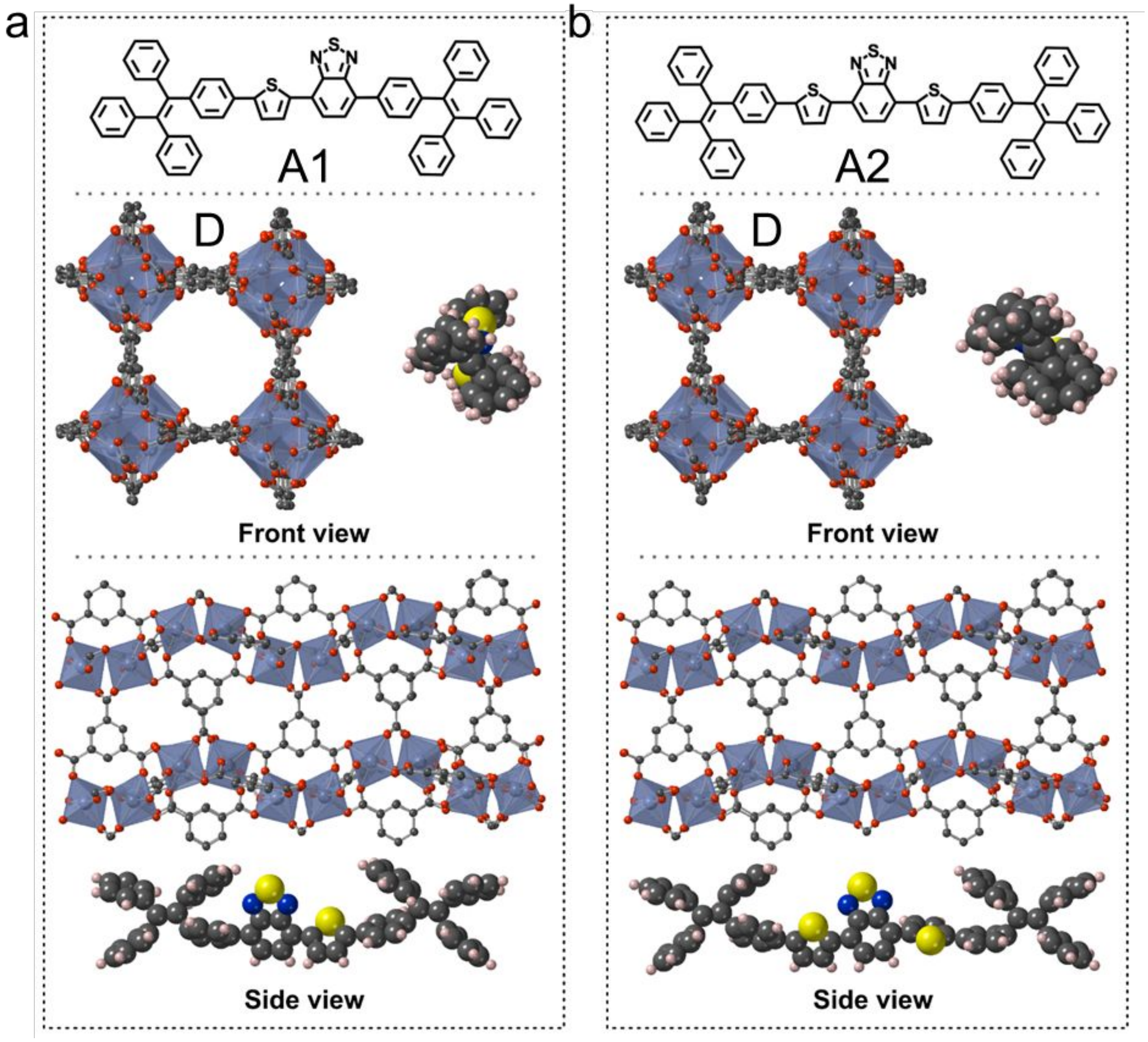

Figure S7. Structure characterization. Schematic showing the molecular structures and geometrical constraints of (a) DA1 and (b) DA2 with A surrounding D. Tb, O, C, N and S atoms are shown in purple, red, grey, blue, and yellow, respectively. H atoms are omitted.

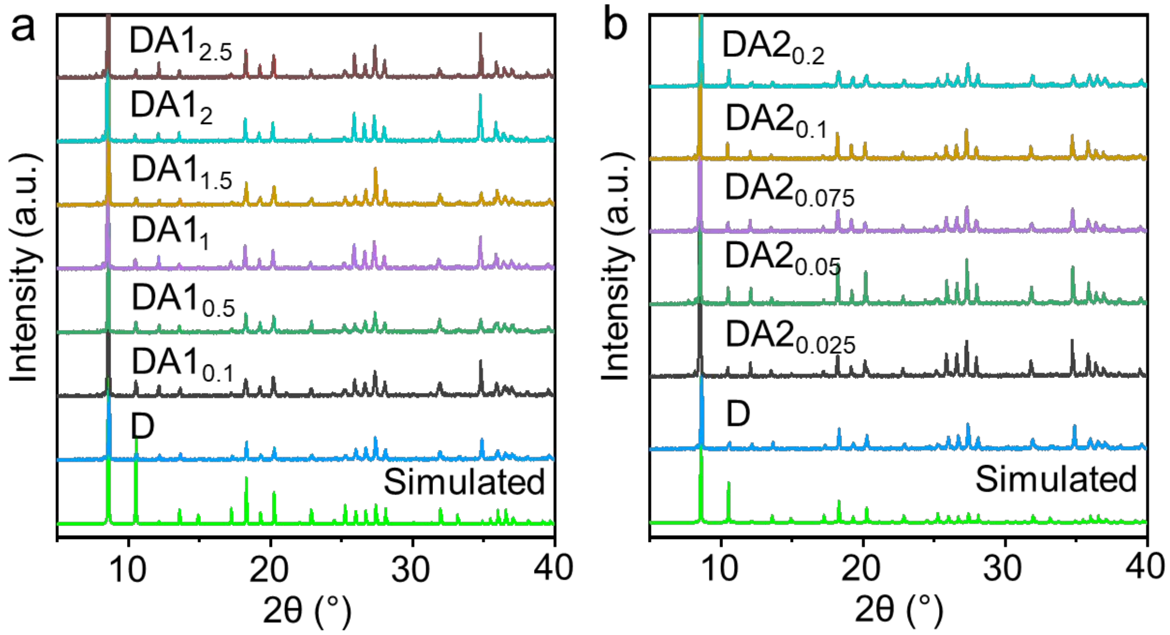

Figure S8. Powder X-ray diffraction (PXRD) patterns of a) DA1$_n$ and b) DA2$_n$ composites (*n* represents the weight percentage (wt%) of A1 or A2, with the concentration of D fixed at 50 wt%) compared with the simulated pattern.

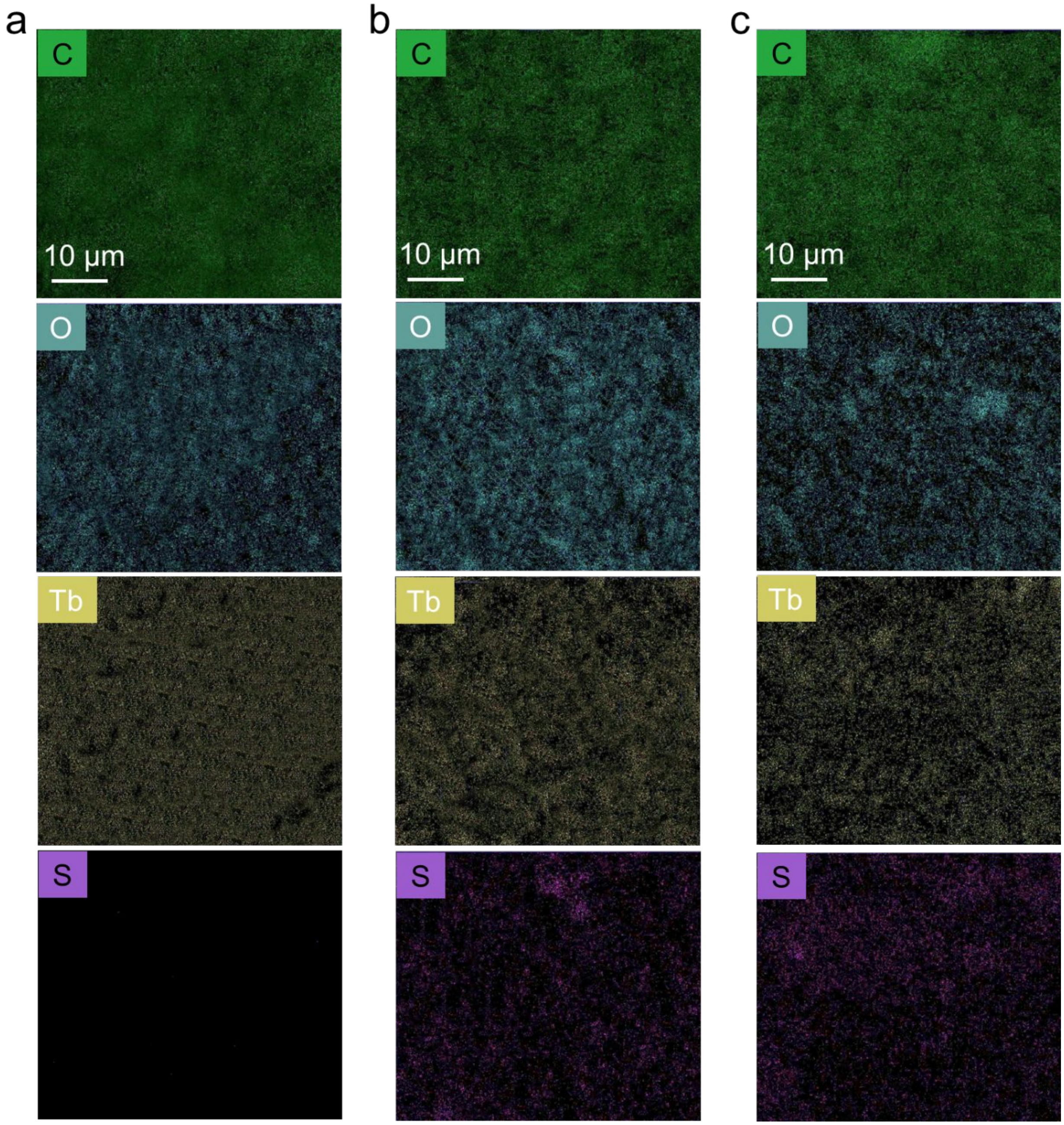

Figure S9. EDS elemental mapping images of C, O, Tb and S elements of (a) Tb-BTC MOF, (b) DA1 and (c) DA2.

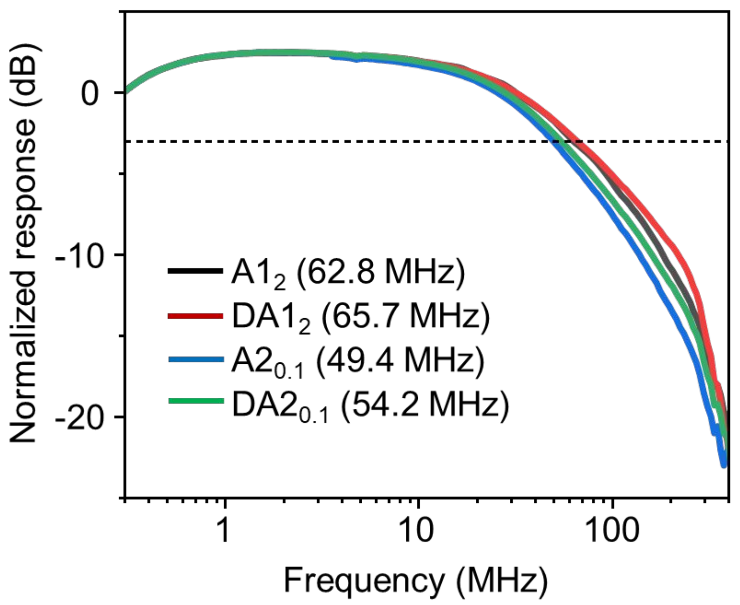

Figure S10. Normalized frequency response of A1, DA1, A2 and DA2.

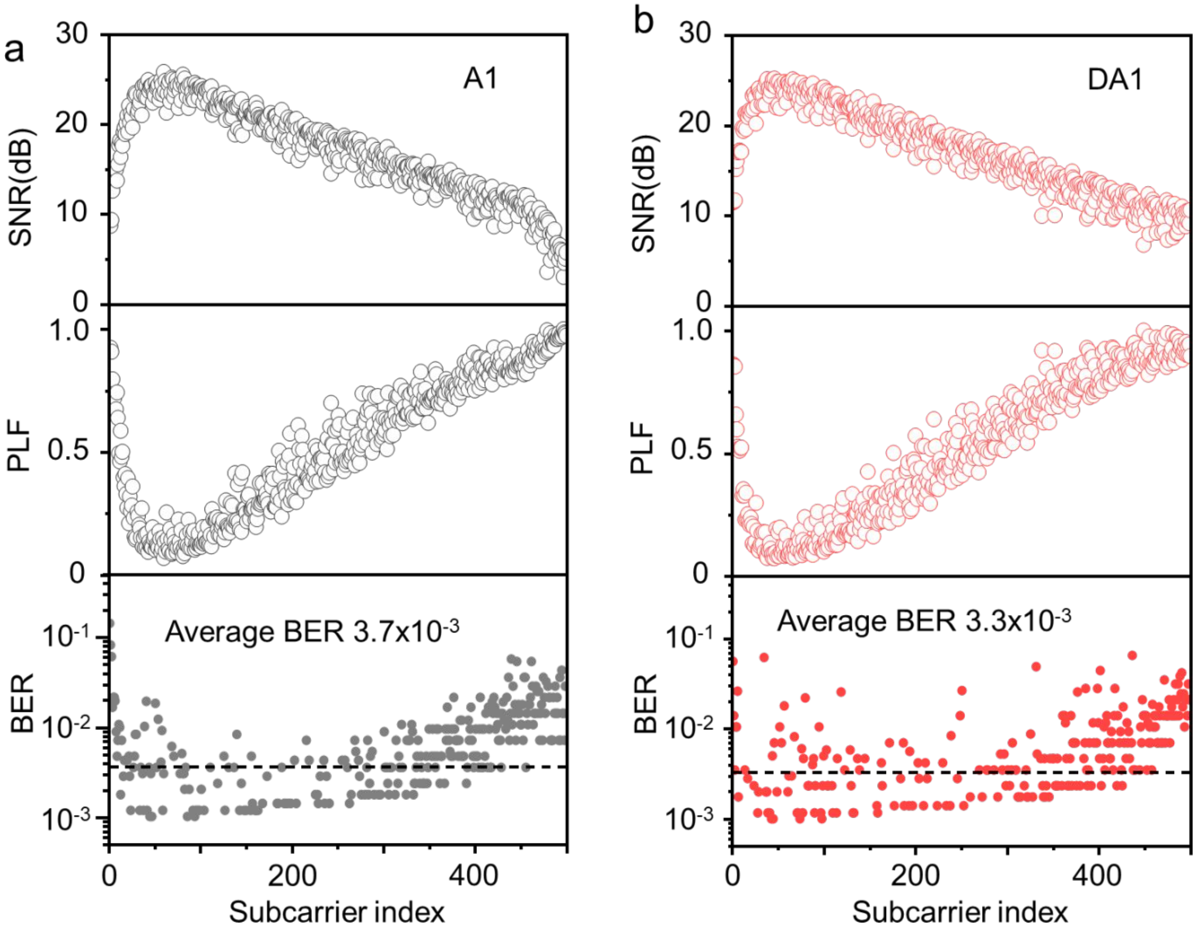

Figure S11. The pertinent metrics for each subcarrier, including estimated signal-to-noise ratio (SNR), power loading factor (PLF), and bit error ratio (BER) of (a) $A1_2$ and (b) $DA1_2$.

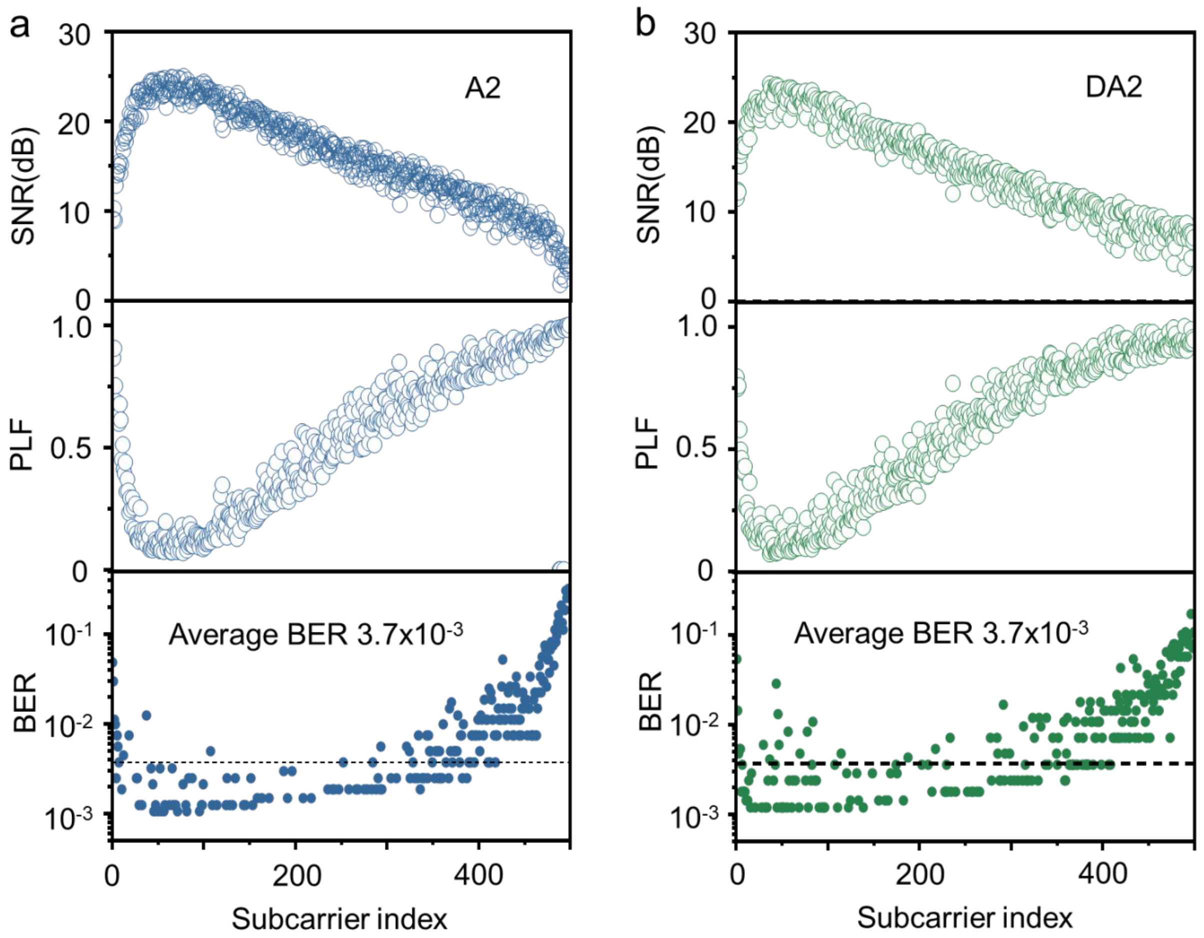

Figure S12. The pertinent metrics for each subcarrier, including estimated signal-to-noise ratio (SNR), power loading factor (PLF), and bit error ratio (BER) of (a) $A2_{0.1}$ and (b) $DA2_{0.1}$.

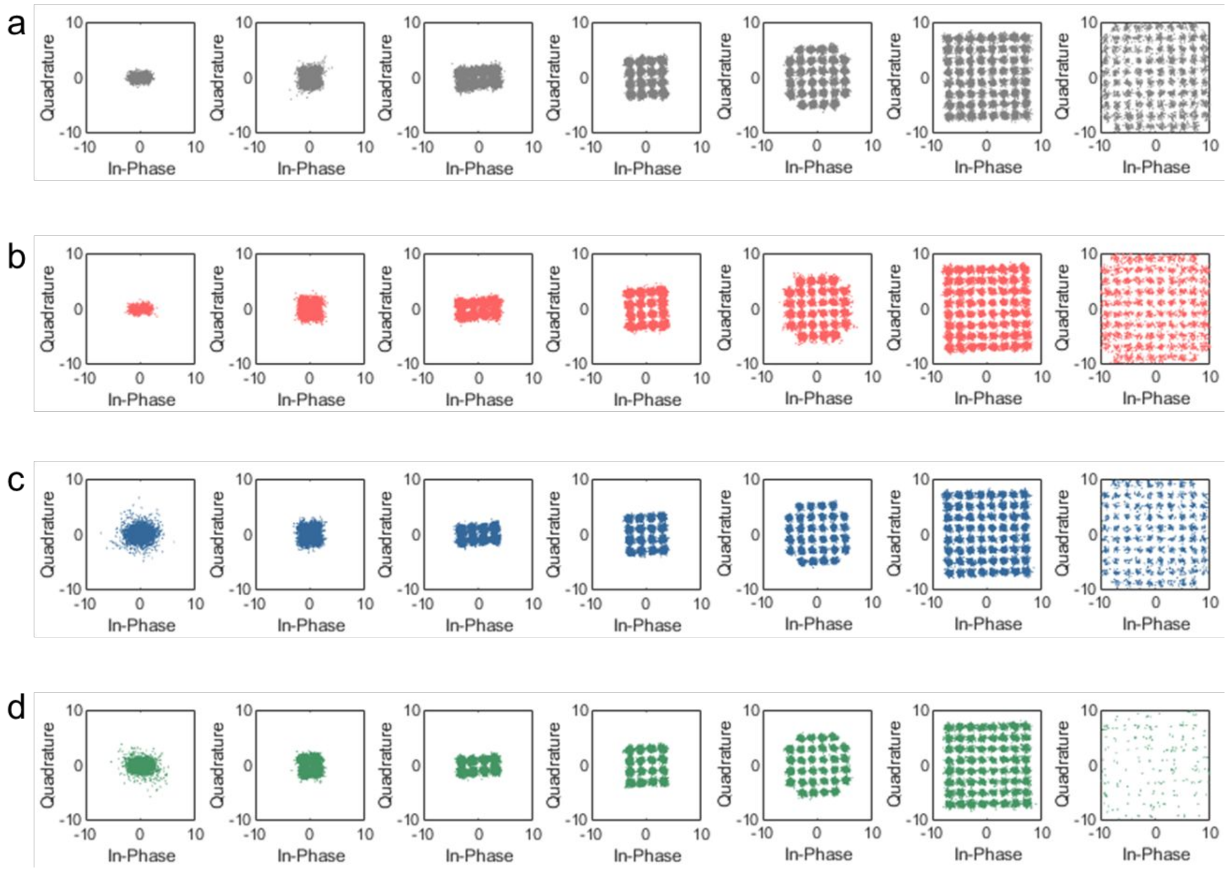

Figure S13. Constellation diagrams of the receiving signal with (a) $A1_2$, (b) $DA1_2$, (c) $A2_{0.1}$, and (d) $DA2_{0.1}$.

Reference

(1) Zhao, Z.; Deng, C.; Chen, S.; Lam, J. W.; Qin, W.; Lu, P.; Wang, Z.; Kwok, H. S.; Ma, Y.; Qiu, H., et al. Full emission color tuning in luminogens constructed from tetraphenylethene, benzo-2,1,3-thiadiazole and thiophene building blocks. *Chem. Commun.* **2011**, *47*, 8847–8849.

(2) Rosi, N. L.; Kim, J.; Eddaoudi, M.; Chen, B.; O'Keeffe, M.; Yaghi, O. M. Rod packings and metal-organic frameworks constructed from rod-shaped secondary building units. *J. Am. Chem. Soc.* **2005**, *127*, 1504–1518.

(3) Kresse, G.; Furthmüller, J. Efficiency of ab-initio total energy calculations for metals and semiconductors using a plane-wave basis set. *Comput. Mater. Sci.* **1996**, *6*, 15–50.
